# Reply to Cirillo: Early-life exposure to the sugar-rationing regime, not a measured sugar dose, drives the cancer gradient

**DOI:** 10.1073/pnas.2632695123

**Published:** 2026-10-30

**Authors:** Chen Zhu, Weilong Zhang

**Affiliations:** ^a^ College of Economics and Management, China Agricultural University, Beijing 100083, China; ^b^ Beijing Food Safety Policy and Strategy Research Base, China Agricultural University, Beijing 100083, China; ^c^ Academy of Global Food Economics and Policy, China Agricultural University, Beijing 100083, China; ^d^ Faculty of Economics, University of Cambridge, Cambridge CB3 9DD, United Kingdom; ^e^ Selwyn College, University of Cambridge, Cambridge CB39DQ, United Kingdom

We thank Cirillo (1) for a close reading of our paper (2) and replication package (3). The comment concerns what the exposure measures rather than the estimates. Read as effects of early-life exposure to the sugar-rationing regime, not of a measured sugar dose, our findings survive each of the four points.

1. Timing. Relaxation of rationing was staged: Sweets were derationed in February 1953, and the sugar ration rose from 10 to 14 oz per week on 19 April and to 16 oz on 17 May, before abolition in September (4). Household purchases also reflect summer stock-building and a postderationing dip (4), not infant ingestion. Redating the cutoff to end June or end March 1953 leaves the gradient intact and changes the hazard ratio per six months by at most 0.02 for every cancer (Table 1).
2. Infant exposure. The recommended addition of sugar to National Dried Milk (5) documents a direct channel from household sugar availability to infant intake. Identification does not require infants under rationing to have consumed no added sugar; it requires the regime and its removal to have shifted sugar availability and, through such channels, infant intake. We agree that our description of infant allocations was too strong: Green-book entitlements existed, initially for the mother’s benefit (6), while individual consumption is unobserved. Because treatment is assigned by policy exposure rather than observed consumption (ref. 2, *SI Appendix*, section S5), our estimates are intention-to-treat effects.
3. Concurrent dietary changes. We reproduce the comment’s Table 1 only by adding series stored in tens of grams to series in grams (3, 7). Harmonizing units reverses the fruit/vegetable change from +8.8% to −3.3% and the meat change from −4.1 to +2.2% (Table 2). Sugar alone accounts for over three quarters of the 100 to 150 kcal energy rise (ref. 2, *SI Appendix*, section S2). Concurrent changes confound only if they vary across cohorts in a way that could generate the exposure-duration gradient. As we note (ref. 2, *SI Appendix*, section S8), sugar and calories cannot be fully separated, and higher energy intake may lie on the pathway from sugar availability to later health rather than confound it.
4. Dose–Response. We agree that the paper reported categorical estimates without a formal trend test. Replacing the exposure groups with a continuous measure, months of the first 1,000 d under rationing (8, 9), gives hazard ratios per six additional months from 0.74 for liver cancer to 0.92 for breast cancer, with every CI excluding 1 (Table 1). Individual point estimates are not perfectly monotonic, particularly for outcomes with few events, but the trend estimates show a systematic relationship between duration under rationing and later cancer risk (departures from linearity: Table 1).

**Table 1.** Trend tests and alternative cutoffs

HR per 6 mo, by cutoff				
Cancer	Sep 1953	Jun 1953	Mar 1953	Departure from linearity, P
Lung	0.88 (0.83 to 0.94)	0.88 (0.82 to 0.94)	0.89 (0.82 to 0.97)	0.194
Liver/IHBD	0.74 (0.64 to 0.84)	0.72 (0.61 to 0.85)	0.74 (0.61 to 0.89)	0.021
Rectum	0.90 (0.85 to 0.96)	0.90 (0.84 to 0.96)	0.89 (0.82 to 0.96)	*<*0.001
Prostate	0.87 (0.84 to 0.89)	0.86 (0.83 to 0.89)	0.87 (0.84 to 0.89)	0.115
Breast	0.92 (0.90 to 0.94)	0.92 (0.90 to 0.95)	0.90 (0.88 to 0.93)	0.038

Each cell is a hazard ratio (95% CI) from a Cox model fitted to UK Biobank data (10) that replaces the five exposure-cohort indicators of ref. 2 with one linear term: months of the first 1,000 d under rationing, expressed per 6 mo. Preperiod indicators and covariates follow ref. 2. Columns redate the end of rationing to 26 September 1953 (published), end June 1953 (after the 17 May ration increase), or end March 1953 (after sweets derationing and the 19 April increase). The last column is a Wald test of whether the five published cohort estimates depart from a linear gradient; a large P indicates no evidence against linearity.

**Table 2.** Change in household purchases, four quarters after versus four quarters before September 1953, from the replication data (3, 7)

Category	As reported (1)	Harmonized
Fats (butter, margarine, lard)	+12.6%	+12.6%
Fruit and vegetables	+8.8%	−3.3%
Dairy (milk, welfare milk, cheese)	+0.2%	−1.2%
Cereals	−0.6%	−3.4%
Meat (with fish, bacon)	−4.1%	+2.2%

“As reported” reproduces Cirillo’s Table 1 by adding series stored in grams to series stored in tens of grams; “harmonized” rescales all series to grams per day. The components of each category are inferred from reproducing the comment’s figures; the fat increase is unaffected by the correction.

Cirillo’s historical evidence clarifies the nature and timing of the exposure. Read as long-term effects of differential early-life exposure to the rationing environment rather than of a precisely measured quantity of sugar, our estimates are not materially altered by the alternative exposure definitions or concurrent dietary changes raised in the comment.

## Data Availability

Some study data are available. UK Biobank data are available to approved researchers by application to UK Biobank (application 89068) (10). The National Food Survey series and the code producing tables 1 and 2 of this reply are deposited in the replication package for ref. 2 at https://github.com/weilongzhang1988/sugar-rationing-PNAS-replication (folder code/reply_to_cirillo) (3).
